# The exploratory behaviour of rats in the hole-board apparatus: Is head-dipping a valid measure of neophilia?

**DOI:** 10.1016/j.beproc.2008.02.019

**Published:** 2008-07

**Authors:** Gillian R. Brown, Christopher Nemes

**Affiliations:** Institute of Behavioural and Neural Sciences, School of Psychology, University of St. Andrews, South Street, St. Andrews, Fife KY16 9JP, UK

**Keywords:** Exploration, Hole-board, Head-dipping, Neophilia, Neophobia, Rat

## Abstract

The exploratory behaviour of laboratory rodents is of interest within a number of areas of behavioural pharmacology. However, how best to measure exploratory behaviour in rodents remains a contentious issue. Many unconditioned tests, such as the open field, potentially confound general locomotor activity with exploration. The hole-board apparatus appears to avoid this confound, as head-dipping into holes in the floor is assumed to be a valid measure of the subject's attraction towards novelty (neophilia). This study aimed to investigate whether head-dipping should be considered a valid measure of neophilia by comparing performance of adult male and female Lister hooded rats on the hole-board task (a) over repeated sessions and (b) when novel objects were absent or present underneath the holes. The results show that head-dipping initially decreased across repeated exposures, while time spent in the aversive central area increased. No change in head-dipping was seen in response to objects being placed underneath the holes. Rather than being a measure of neophilia, these results support the hypothesis that head-dipping represents an escape response, which declines as the subject becomes less fearful. These results are compared with previous studies of repeated exposure to other novel environments.

## 1 Introduction

When faced with an unfamiliar environment or object, animals often exhibit behaviour patterns that broadly can be termed exploration, such as locomoting around the environment, orientating towards novelty, and touching or sniffing novel objects (Berlyne, 1950, 1960; Glickman and Sroges, 1966; Welker, 1957). Exploration potentially provides an animal with new information about food sources, shelters or mating opportunities. However, by entering a new environment or attending to a novel stimulus, an animal might also increase it's risk of predation, aggression from conspecifics or other hazards. Whether an animal investigates or avoids novelty has been described as the outcome of an approach–avoidance conflict (Montgomery, 1954, 1955; Montgomery and Monkman, 1955) or as a balance between neophilic and neophobic tendencies (Greenberg, 2003). In motivational terms, neophilia can be defined as the attraction that an animal displays towards an object or place simply because it is novel, while neophobia is the aversion that an animal shows towards approaching a novel object or place (Greenberg, 2003). In behavioural terms, neophilia and neophobia can be considered respectively as curiosity-based approach to, and fear-based avoidance of, a novel stimulus (Hughes, 2007).

The exploratory behaviour of rodents has gained recent interest within a number of areas of behavioural pharmacology. For instance, researchers studying drug addiction are interested in the neural mechanisms underlying neophilia due to the apparent overlap with the neural mechanisms involved in the rewarding effects of drug-taking (Bardo et al., 1996). However, considerable controversy still surrounds the question of how best to measure neophilic and neophobic responses in laboratory animals. One of the most commonly used behavioural tests for laboratory rodents is known as the open field. Originally, the open field apparatus consisted of a flat, raised platform (Hall, 1934, 1936), although the term open field is now commonly used to refer to any enclosed arena that can range in size from a small box to a large playing field (Crawley, 1985; Whishaw et al., 2006). In such an arena, the overall level of locomotion and time spent in the centre of the arena (which is assumed to be aversive to rodents) are often interpreted as measures of exploratory behaviour.

However, some researchers have argued that forcing an animal to be in an enclosed area, or on an open platform, does not allow the animal to exhibit its ‘motivation’ to explore an unknown environment, as the task evokes a strong fear response (Birke and Sadler, 1986; Denenberg, 1969; Renner, 1990; Walsh and Cummins, 1976). Corticosterone levels have been found to rise in rodents on exposure to a novel open field environment (e.g. Marin et al., 2007; Matzel et al., 2006; Rees et al., 2006), and open field behaviour is influenced by some anxiolytic (anxiety-reducing) substances (Prut and Belzung, 2003). In particular, benzodiazepines and serotonin receptor agonists, which have anxiolytic effects in human beings, generally increase the proportion of entries into the centre of the open field in rodents (Prut and Belzung, 2003). Together, these data suggest that the open field may provide valid behavioural measures of fearfulness, but may have limited value for researchers interested in measuring neophilia. Another common concern with interpreting open field tests is that differences in the performance of animals in the open field may result simply from differences in overall locomotor activity, which could be unrelated to differences in exploratory behaviour (Berlyne, 1960; Birke and Archer, 1983).

In the 1970s, researchers began to use the *hole-board* apparatus, which consists of an enclosed arena with holes in the floor into which an animal can poke it's head, referred to as head-dipping (e.g. File and Wardill, 1975a,b; Nolan and Parkes, 1973). The frequency and duration of head-dipping are assumed to provide measures of neophilia (or directed exploration) that are independent from the general locomotor activity of the animal (File and Wardill, 1975a; Ljungberg and Ungerstedt, 1976). This apparatus has been argued, therefore, to avoid the difficulties of interpreting general locomotion that prove problematic in the open field, and a number of studies have shown that head-dipping and locomotion can vary independently of each other (e.g. Abel, 1995; Durcan and Lister, 1989; File, 1977; Lister, 1987; Rogers et al., 1999). In general, high levels of head-dipping are interpreted as indicative of neophilia, while low levels are assumed to result from a lack of neophilia or are assumed to reflect a high anxiety-like state in the animal (Crawley, 1985; Takeda et al., 1998). The hole-board task is currently being used as a test of neophilia in many areas of behavioural pharmacology (Kliethermes and Crabbe, 2006a).

Researchers have attempted to validate head-dipping as a measure of neophilia by administering different classes of drugs and by comparing different genetic strains of rodents in their performance on the hole-board task. For example, if head-dipping is a neophilic response that is suppressed by an anxiety-like response, treatment with anxiolytic agents is predicted to increase head-dipping. Such studies have produced conflicting evidence; for instances, treatment of rodents with anxiolytic benzodiazepines has been reported to increase (rats: File, 1977; mice: Nolan and Parkes, 1973; Takeda et al., 1998), decrease (rats: Pellow et al., 1985) or have no effect (rats: Sayin et al., 1992) on the frequency of head-dipping. A recent review has suggested that the effects of anxiolytic compounds on head-dipping behaviour are generally confounded by changes in overall locomotion, despite the claims that head-dipping is unrelated to locomotor activity (Kliethermes and Crabbe, 2006a). Similarly, a study of several inbred mouse strains reported that head-dipping and locomotion are highly correlated (Kliethermes and Crabbe, 2006b). Whether head-dipping can be interpreted as a valid measure of neophilia remains unresolved (Bilkei-Gorzó and Gyertyán, 1996; Renner, 1990).

The aim of this study was to investigate whether head-dipping behaviour should be considered a valid measure of neophilia by comparing performance of rats on the hole-board task (a) over repeated sessions and (b) when objects are placed underneath the holes. Repeated exposure to a novel apparatus is expected to produce a reduction in exploration as the animal becomes familiar with the environment, a process commonly referred to as habituation (Leussis and Bolivar, 2006). If head-dipping behaviour is a measure of neophilia, the frequency of head-dipping is therefore predicted to decrease over repeated sessions. In early study by Nolan and Parkes (1973), head-dipping by young mice (21–25 postnatal days) was reported to be lower on a second exposure to the hole-board apparatus compared to the first exposure. Two recent studies have also provided evidence that head-dipping by mice and rats decreases on repeated exposure to the hole-board apparatus (Gagliano et al., 2008; Mayeux-Portas et al., 2000). If this behaviour is a valid measure of neophilia, head-dipping is also predicted to be higher in the presence, than in the absence, of objects. Although an early study reported that rodents head-dip more frequently when objects are present (File and Wardill, 1975a), this finding has not received recent replication. Given that male and female rodents are reported to exhibit behavioural differences on the hole-board task (e.g. Aguilar et al., 2003; Ray and Hansen, 2004), subjects of both sexes were included.

## 2 Materials and methods

### 2.1 Subjects and housing

The subjects of this experiment were eight male and eight female adult Lister Hooded rats (supplied by Harlan, U.K.). The animals were housed in a single room, which was controlled for temperature and humidity and was maintained on a 12-h light:dark cycle (lights on at 07:00). The animals were housed in same-sex pairs in plastic and wire mesh home-cages (measuring 25 cm × 45 cm × 15 cm) with *ad libitum* access to rodent pellets and water. All guidelines and requirements set out in the Principles of Laboratory Animal Care (National Institutes of Health, U.S.A., Publication No. 86-23, revised 1985) and the U.K. Animals (Scientific Procedures) Act 1986 were followed.

### 2.2 Apparatus and experimental design

The hole-board apparatus consisted of a wooden, grey box, measuring 68 cm × 68 cm. The walls were 40 cm high, and the box was raised 28 cm above the ground on a metal stand. Four holes (4 cm in diameter) were cut into the floor of the apparatus; each hole was 28 cm from a corner of the box along the diagonal from the corner to the centre. The floor of the box was marked out into four outer areas and one central area using black masking tape. The central area was delineated by four lines of tape each 20 cm from one of the walls, while the four outer areas were marked out by diagonal lines of tape running from the corners of the floor to the corners of the central square. The four holes were thus located at the corners of the central square. The apparatus was located in a small testing room with dimmed white lighting. The stand of the apparatus was open on all sides, allowing the floor or objects to be dimly lit.

Each subject was tested ten times in the hole-board apparatus, once per day during two sets of five consecutive days (Monday–Friday and the following Monday–Friday). During the first set of five trials, no objects were present underneath the holes of the apparatus; during the second set of five trials, an object was placed on the floor under each of the four holes prior to the start of the trial, approximately 20 cm below each hole. The objects were all distinct from each other but were similar in size (approximately 10 cm in length or diameter: a black-and-white rubber ball, a purple plastic star, a red-and-white rubber pet toy, and a yellow, rubber dumb-bell shaped pet toy).

All trials were carried out between 09:00 and 17:00 h, and trials on males and females were alternated throughout the day. At the beginning of each trial, a subject was placed in one corner of the apparatus (always the corner closest to the door of the room), facing the centre of the arena. Each trial lasted 10 min. At the end of the trial, the subject was immediately placed into a carrying box and returned to the home cage. Between each trial, the floor and walls of the apparatus and the novel objects, if present, were cleaned with 70% alcohol solution.

### 2.3 Behavioural measurements

During each 10-min trial, behavioural data were recorded by the observer (C.N.) onto a spreadsheet that was divided into 60 10-s time blocks. Inter-observer reliability between two independent observers (C.N. and G.R.B.) was over 80%. The following behaviour patterns were recorded:

(i) enter a new area: the animal moves from one area of the open field to another (all four paws had to be placed on the floor of a new area);
(ii) head-dip: the animal places it's head into one of the holes, to a minimum depth such that the ears were level with the floor of the apparatus (a new bout of head-dipping was recorded if the animal raised it's head fully out of the hole before resuming);
(iii) rear: the animal is stationary on it's backpaws and raises it's forepaws off the ground, extending it's body vertically.

The data on entries into a new area were used to calculate the total amount of locomotion (number of entries into all areas summed together) and the percentage of entries that were in made into the central area. The location of the animal during each of the 10-s time intervals was used to estimate the percentage of time spent in the central area.

### 2.4 Statistical analyses

The data were analysed using repeated-measure ANOVAs, with ‘objects’ (with or without) as a within-subject variable, ‘trial’ (trials 1–5) as a within-subject repeated measure and ‘sex’ (male or female) as a between-subject variable. Significant interactions were analysed further using simple effects *post hoc* tests (Howell, 2007).

## 3 Results

### 3.1 Total locomotion

The total amount of locomotion did not vary across trials (*F*_4,56_ = 0.096, n.s.) or vary with the presence or absence of objects (*F*_4,56_ = 0.071, n.s.). Females locomoted more on average than males (*F*_1,14_ = 4.871, *p* = 0.045; Table 1). There were no interactions between trial and sex (*F*_4,56_ = 1.433, n.s.), trial and object (*F*_4,56_ = 2.615, n.s.) or object and sex (*F*_4,56_ = 0.559, n.s.), and the three-way interaction between these variables was not significant (*F*_4,56_ = 1.174, n.s.).

### 3.2 Locomotion into the central area

**Fig. 1:**
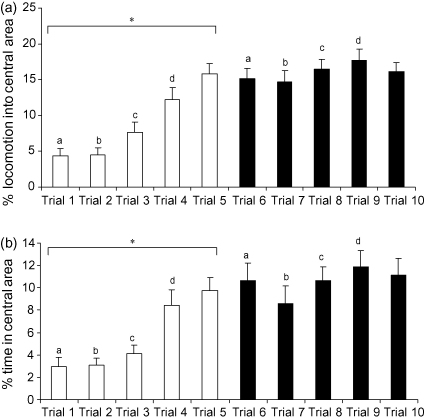
(a) Percentage of entries into the central area across five trials without objects (white bars) and five trials with objects (black bars) (means and S.E.M.; *N* = 16). *Post hoc* analyses: *p < 0.01; a–d represent pairs of trials that differ at *p* < 0.01; (b) percentage of time spent in the central area across five trials without objects (white bars) and five trials with objects (black bars) (means and S.E.M.; *N* = 16). *Post hoc* analyses: *p < 0.01; a–d represent pairs of trials that differ at *p* < 0.01.

The percentage of entries that were into the central area differed significantly across trials (*F*_4,56_ = 14.842, *p* < 0.001) and varied with the presence or absence of objects (*F*_1,56_ = 84.240, *p* < 0.001). There was also a significant interaction between trials and the presence or absence of objects (*F*_4,56_ = 7.662, *p* < 0.001). *Post hoc* analyses indicated that the proportion of entries into the centre increased over the first five trials and remained steady thereafter (Fig. 1a). The percentage of entries into the central area varied between the sexes (*F*_1,14_ = 5.209, *p* = 0.039), with females exhibiting an overall greater proportion of entries into the centre than males (Table 1). There were no interactions between trial and sex (*F*_4,56_ = 0.761, n.s.) or object and sex (*F*_4,56_ = 1.956, n.s.), and the three-way interaction between these variables was not significant (*F*_4,56_ = 2.567, n.s.).

### 3.3 Time spent in the central area

The percentage of time spent in the central area differed significantly across trials (*F*_4,56_ = 8.085, *p* < 0.001) and varied with the presence or absence of objects (*F*_1,56_ = 35.393, *p* < 0.001). There was also a significant interactions between trials and the presence or absence of objects (*F*_4,56_ = 3.493, *p* = 0.013). Further analyses indicated that the time spent in the centre increased over the first five trials and remained steady thereafter (Fig. 1b). The percentage of time spent in the central area varied between the sexes (*F*_1,14_ = 5.032, *p* = 0.042), with females exhibiting an overall greater percentage of time in the centre than males (Table 1). There were no interactions between trial and sex (*F*_4,56_ = 0.083, n.s.) or object and sex (*F*_4,56_ = 0.076, n.s.), and the three-way interaction between these variables was not significant (*F*_4,56_ = 1.001, n.s.).

### 3.4 Frequency of head-dipping

**Fig. 2:**
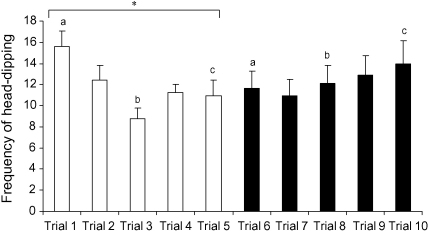
Frequency of head-dipping during five trials without objects (white bars) and five trials with objects (black bars) (means and S.E.M.; *N* = 16). *Post hoc* analyses: *p < 0.01; a–c represent pairs of trials that differ at *p* < 0.01.

The frequency of head-dipping differed significantly across trials (*F*_4,56_ = 2.626, *p* = 0.043), and there was a significant interaction between trials and the presence or absence of objects (*F*_4,56_ = 4.482, *p* = 0.013). Further analyses indicate that head-dipping decreased over the first five trials and slightly increased towards the end of the experiment (Fig. 2). There was no main effect of the presence or absence of objects (*F*_1,14_ = 0.142, n.s.). The frequency of head-dipping varied significantly between the sexes (*F*_1,14_ = 15.401, *p* = 0.002), with females exhibiting an overall greater frequency of head-dipping than males (Table 1). There were no interactions between trial and sex (*F*_4,56_ = 0.324, n.s.) or object and sex (*F*_4,56_ = 0.553, n.s.), or a three-way interaction between these variables (*F*_4,56_ = 0.779, n.s.).

### 3.5 Frequency of rearing

**Fig. 3:**
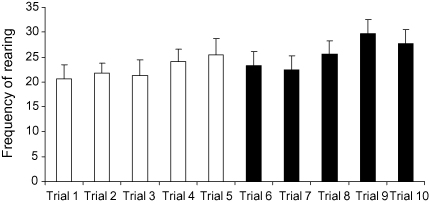
Frequency of rearing during five trials without objects (white bars) and five trials with objects (black bars) (means and S.E.M.; *N* = 16).

The frequency of rearing differed significantly across trials (*F*_4,56_ = 3.204, *p* = 0.019), with frequency increasing slightly across sessions (Fig. 3). There was no main effect of the presence or absence of objects (*F*_1,14_ = 3.898, n.s.) or sex (*F*_1,14_ = 1.332, n.s.; Table 1). There were no interactions between trials and the presence or absence of objects (*F*_4,56_ = 0.457, n.s.), between trial and sex (*F*_4,56_ = 0.111, n.s.) or object and sex (*F*_4,56_ = 0.006, n.s.), or a three-way interaction between these variables (*F*_4,56_ = 1.708, n.s.).

## 4 Discussion

The aim of this study was to investigate whether head-dipping behaviour should be considered a valid measure of neophilia by comparing performance on the hole-board task (a) over repeated sessions (trials 1–10) and (b) when no objects were present (trials 1–5) and when objects were placed underneath the holes (trials 6–10). The results show that head-dipping was high during the first test, decreased over the following two trials and remained relatively stable during the rest of the experiment. The initial drop in head-dipping following the first trial could be interpreted in two ways. First, head-dipping could be indicative of a neophilic response that decreases as the animal becomes familiar with the apparatus, i.e. head-dipping represents directed exploratory behaviour that drops as the apparatus loses its novelty. If this interpretation is correct, we would also predict that head-dipping would be greater in the presence of objects; however, there was no evidence of an increase in head-dipping behaviour when objects were present underneath the holes. These results do not support the hypothesis that head-dipping is a valid measure of neophilia.

The second interpretation of the initial drop in head-dipping frequency is that head-dipping could represent a fearful, neophobic response, such that, on first exposure to the apparatus, the animal actively attempts to find an escape route (Renner, 1990). Adult male rats have been shown to exhibit an increase in circulating corticosteroid levels following a single exposure to the hole-board apparatus (Márquez et al., 2005, 2006), suggesting that testing in this apparatus is a stressful event. If this interpretation of head-dipping is correct, we would also predict that, as head-dipping behaviour declines, fearfulness would also decline. In favour of this interpretation, while head-dipping frequency declined over the first few tests, the amount of locomotion into the central area of the hole-board, and the time spent in the central area, greatly increased over these trials. Therefore, as fearfulness apparently decreased, head-dipping also decreased. If we assume that the fear experienced on exposure to a novel apparatus can be equated to normal or ‘state’ anxiety (Belzung and Griebel, 2001), these results contradict the assumption that head-dipping behaviour is suppressed by an anxiety-like response, in which case we might have expected head-dipping to vary in the opposite direction to anxiety-like behaviour.

A small number of previous studies on rodents have also presented evidence that the frequency of head-dipping decreases on second exposure to the hole-board apparatus (Gagliano et al., 2008; Mayeux-Portas et al., 2000; Nolan and Parkes, 1973). The early study by Nolan and Parkes (1973) investigated whether previous experience with the hole-board apparatus with a solid floor inserted would influence frequency of head-dipping on second exposure to the apparatus. The results indicated that prior exposure to the plain board reduced the frequency of head-dipping on subsequent exposure as much as prior exposure to the board with holes (Nolan and Parkes, 1973). Again, these results do not support the idea that head-dipping provides a valid measure of neophilia, rather than a neophobic response induced by an unfamiliar test situation.

In our study, head-dipping did increase in frequency towards the end of the experiment, after eight or more exposures to the apparatus. Therefore, we cannot reject the possibility that, as the subjects became very familiar with the apparatus, they engaged in a greater level of visual exploration through the holes. Rearing behaviour is also commonly interpreted as an activity by which an animal obtains information about distal environmental cues (Lever et al., 2006). Given that rearing behaviour gradually increased in frequency across the sessions, as the subjects presumably became less fearful, the amount of visual assessment of the testing room, both above and below the apparatus, may have increased. This interpretation is supported by an experiment carried out by Bilkei-Gorzó and Gyertyán (1996). This study reported that the effects of the anxiolytic benzodiazepine chlordiazepoxide on head-dipping behaviour in rats varied with the light intensity during testing, such that treatment decreased head-dipping in very bright light and increased head-dipping under normal light. The authors argued that head-dipping during the aversive testing condition (bright light) represents an attempt by the subject to find an escape route from the apparatus, while head-dipping in less aversive conditions represents visual exploration of the apparatus. Future studies could investigate whether head-dipping increases with extended familiarisation to the apparatus even in the absence of novel objects, and whether head-dipping increases when novel objects are brought closer to the holes, when new novel objects are placed under the holes for each test, or when objects are re-located beneath different holes.

Our results show that repeated exposure of subjects to a novel hole-board apparatus greatly affects the behavioural response, and that the neophobic response experienced by subjects during the first exposure to an apparatus apparently declines over further exposures. Repeated exposure to another commonly used behavioural test, the elevated maze, has been reported to result in a decrease in total locomotion and a decrease in the amount of time spent on aversive open sections of the maze (e.g. Cook et al., 2002; Dawson et al., 1994; Rodgers et al., 1996). In contrast, in our study, no change of overall locomotion was found on repeated exposure to the hole-board, and the amount of time spent in the aversive central area of the hole-board apparatus increased over sessions. The reason for the differences in the behavioural effects of repeated exposure to these two types of novel environments may be related to differences in the design of the two types of apparatus. Repeated exposure to an elevated maze, which consists of open areas and closed areas, may have resulted in the subjects retreating into the darker, closed arms once an initial investigation had found no potential escape routes from the apparatus; such a response was not an option in the hole-board test. If a shelter were added to the hole-board apparatus, locomotion might be predicted to decrease over repeated exposure, with subjects choosing to remain under a shelter once potential escape routes had been investigated.

In support of previous reports (e.g. Aguilar et al., 2003; Ray and Hansen, 2004), our results indicate that female rats head-dip more frequently, locomote more and spend more time in the centre of the hole-board apparatus than males. These sex differences in behaviour were not affected by repeated exposure to the apparatus, or by the presence of novel objects. Given that the locomotor patterns of female rodents are influenced by the stage of the estrus cycle and by experimental manipulation of hormones such as estradiol (e.g. Garey et al., 2001; Morgan and Pfaff, 2002), researchers have questioned whether gonadal hormones might influence general levels of arousal that might impact on how females respond to novelty compared to males (Morgan et al., 2004). However, while reports of sex differences in hole-board performance have been interpreted as showing that females are more exploratory than males, females have been reported to locomote more than males in familiar, as well as novel, environments (e.g. Cortright et al., 1997; Eckel and Moore, 2004), and, as discussed above, head-dipping may not represent neophilic behaviour. Therefore, the conclusion that females are more exploratory or neophilic than males should not be drawn from such data.

The results of this study indicate that the assumption that head-dipping on the hole-board task represents a neophilic response is not necessarily accurate. At least on first exposure to the apparatus, head-dipping is likely to result from an attempt by the subject to find a potential escape routes. This issue highlights a more general problem with unconditioned behavioural tests of exploratory behaviour, in that high levels of locomotion around an environment should not immediately be interpreted as neophilia or a positively rewarded aspect of novelty-seeking (Welker, 1957). Any behaviour in a novel environment will be influenced by both neophilia and neophobia, such that a single behavioural measure is unlikely to be purely indicative of either neophilic or neophobic tendencies. Rather than being at polar ends of a continuum, neophilia and neophobia should be thought of as two orthogonal factors that can vary independently (Greenberg, 2003). For researchers interested in rodent exploration, simple measures, such as head-dipping, are unlikely to expose by themselves the complex interaction between these two factors.

## Figures and Tables

**Table 1:** Means + S.E.M. or behavioural measures for males and females in the hole-board apparatus

	Without objects (trials 1–5)	With objects (trials 6–10)	Mean (trials 1–10)
Total locomotion			
Males	50.6 ± 2.7	49.5 ± 4.2	50.0 ± 3.3
Females	57.5 ± 2.6	59.9 ± 3.1	58.7 ± 2.2*

% entries into centre			
Males	6.6 ± 1.3	14.8 ± 1.5	10.73 ± 1.2
Females	11.2 ± 1.1	17.2 ± 1.0	14.2 ± 0.9*

% time in the centre			
Males	4.1 ± 0.9	8.8 ± 1.4	6.5 ± 1.1
Females	7.2 ± 0.8	12.3 ± 1.5	9.8 ± 1.0*

Frequency of head-dipping			
Males	9.3 ± 1.1	8.8 ± 1.8	9.1 ± 1.0
Females	14.3 ± 0.5	15.8 ± 1.9	15.0 ± 1.1*

Frequency of rearing			
Males	20.3 ± 11.1	23.5 ± 8.4	21.9 ± 9.0
Females	25.0 ± 5.6	28.0 ± 8.5	26.5 ± 6.9

*p < 0.05 for main effect of sex.
